# Morphological and Perfusion Changes of the Macula Following Different Endotamponade Methods after Retinal Detachment Surgery

**DOI:** 10.22336/rjo.2026.57

**Published:** 2026

**Authors:** Doniyor Sadulla o'g'li Bobojonov, Azamat Farhadovich Yusupov

**Affiliations:** 1 Republican Specialized Scientific and Practical Medical Center of Eye Microsurgery, Tashkent, Uzbekistan

**Keywords:** retinal detachment, endotamponade, optical coherence tomography, OCT angiography, macular perfusion, vitreoretinal surgery, RRD = rhegmatogenous retinal detachment, SF6 = sulfur hexafluoride, C3F8 = perfluoropropane, OCT = optical coherence tomography, OCTA = optical coherence tomography angiography, CMT = central macular thickness, ERM = epiretinal membrane, FAZ = foveal avascular zone, BCVA = best-corrected visual acuity, logMAR = logarithm of the minimum angle of resolution, ANOVA = analysis of variance, SPSS = Statistical Package for the Social Sciences

## Abstract

**Purpose:**

To evaluate and compare the morphological and perfusion changes of the macula following different endotamponade methods in patients undergoing retinal detachment repair surgery.

**Methods:**

A retrospective cohort study of 120 patients with rhegmatogenous retinal detachment who underwent pars plana vitrectomy with different endotamponade agents between January 2022 and May 2025. We divided patients into three groups: silicone oil (n=60), sulfur hexafluoride (SF6) gas (n=38), and perfluoropropane (C3F8) gas (n=22). We assessed macular morphology using optical coherence tomography (OCT) and evaluated perfusion parameters with OCT angiography (OCTA) at baseline and at 1, 3, 6, and 12 months postoperatively. Primary outcomes included central macular thickness (CMT), macular volume, and perfusion density measurements.

**Results:**

Mean follow-up was 14.2 ± 3.8 months. At 12 months, the silicone oil group had significantly greater CMT (287.4 ± 42.3 μm) than SF6 (251.8 ± 28.9 μm, p<0.01).

**Discussion:**

Eyes treated with silicone oil showed persistent macular thickening, a higher rate of epiretinal membrane formation (23.3% vs. 7.9% and 9.1%), lower superficial and deep capillary plexus perfusion density, and a larger foveal avascular zone at 12 months than both gas groups. These structural and microvascular differences paralleled the poorer visual recovery in the silicone oil group (logMAR 0.47 vs. 0.34 and 0.31). Possible mechanisms include inflammatory and toxic effects of prolonged silicone oil contact, blood-retinal barrier disruption, and mechanical effects on the macular microcirculation. These associations should be interpreted with caution because the tamponade was not randomized, and silicone oil was more often used in complex cases.

**Conclusions:**

Different endotamponade agents affect macular morphology and perfusion differently following retinal detachment repair. Gas tamponades show better outcomes in macular thickness normalization and perfusion recovery than silicone oil, which may affect long-term visual outcomes.

## Introduction

Rhegmatogenous retinal detachment (RRD) is a sight-threatening condition requiring prompt surgical intervention to restore anatomical integrity and preserve visual function [1]. Pars plana vitrectomy with endotamponade is the gold-standard treatment, with various tamponade agents available, including silicone oil and expandable gases such as sulfur hexafluoride (SF6) and perfluoropropane (C3F8).

The choice of endotamponade agent can influence surgical outcomes, with each option presenting distinct advantages and limitations. Silicone oil provides longer-lasting tamponade and better postoperative positioning compliance, while gas tamponades offer temporary support with spontaneous absorption, avoiding the need for later removal [2,3].

Recent improvements in optical coherence tomography (OCT) and OCT angiography (OCTA) have enabled more detailed assessment of macular morphology and microvascular perfusion, providing valuable insights into pathophysiological changes following retinal reattachment surgery [4]. Understanding these changes helps optimize treatment strategies and predict long-term visual outcomes.

In this study, we aimed to evaluate macular morphology and perfusion changes after different endotamponade methods, using advanced imaging techniques to quantitatively analyze postoperative macular recovery patterns.

## Methods

### Study design and participants

This retrospective cohort study was carried out at a tertiary Republican Specialized Scientific and Practical Medical Center of Eye Microsurgery (Tashkent, Uzbekistan) between January 2022 and May 2025. The institutional review board approved the study protocol, and the study adhered to the Declaration of Helsinki. We obtained informed consent from all participants.

#### Inclusion criteria

- Primary rhegmatogenous retinal detachment involving the macula;
- Age 18-75 years;
- Clear ocular media allowing adequate OCT imaging;
- Minimum follow-up period of 12 months;
- Successful anatomical reattachment at 1 month postoperatively.

#### Exclusion criteria

- Previous vitreoretinal surgery;
- Diabetic retinopathy or other retinal vascular diseases;
- Macular holes or epiretinal membranes present preoperatively;
- Proliferative vitreoretinopathy grade D;
- Inability to maintain required postoperative positioning.

### Surgical technique

Experienced vitreoretinal surgeons performed all surgeries using standardized 25-gauge pars plana vitrectomy systems. The surgical approach included core vitrectomy, identification and treatment of retinal breaks, endolaser photocoagulation, and fluid-air exchange. Surgeons chose the endotamponade agent based on surgeon preference, break characteristics, and patient factors.

#### Endotamponade groups

1. **Silicone oil group (n=60):** 1000 centistoke silicone oil;
2. **SF6 gas group (n=38):** 20% sulfur hexafluoride;
3. **C3F8 gas group (n=22):** 14% perfluoropropane.

### Imaging protocol

We performed high-resolution OCT imaging using Triton OCT (Topcon, Japan) in enhanced depth imaging mode. We performed OCTA on the same platform using 6×6 mm scan patterns centered on the fovea.

#### Morphological parameters

- Central macular thickness (CMT);
- Macular volume (6 mm diameter);
- Presence of intraretinal/subretinal fluid;
- Epiretinal membrane formation;
- Photoreceptor layer integrity.

#### Perfusion parameters

- Superficial capillary plexus density;
- Deep capillary plexus density;
- Foveal avascular zone area;
- Perfusion density in 3×3 mm central area.

### Statistical analysis

We performed statistical analyses using SPSS version 28.0 (IBM Corporation, Armonk, NY). We expressed continuous variables as mean ± standard deviation, and categorical variables as frequencies and percentages. We used one-way ANOVA to compare continuous variables between groups, followed by post hoc Tukey’s test for pairwise comparisons. We used the chi-square test to compare categorical variables between groups. We used repeated-measures ANOVA to analyze changes over time. We considered p < 0.05 statistically significant.

## Results

### Patient demographics

A total of 120 patients met the inclusion criteria and were included in our analysis. The mean age was 58.7 ± 12.4 years, with 70 males (58.3%) and 50 females (41.7%). Baseline characteristics were similar between groups, with no significant differences in age, gender, lens status, or extent of retinal detachment (p>0.05 for all comparisons).

**Table 1:** Baseline patient characteristics

Parameter	Silicone Oil (n=60)	SF6 Gas (n=38)	C3F8 Gas (n=22)	p-value
Age (years)	59.3 ± 11.8	58.4 ± 13.2	58.2 ± 12.1	0.821
Male gender, n (%)	35 (58.3)	22 (57.9)	13 (59.1)	0.989
Phakic eyes, n (%)	40 (66.7)	25 (65.8)	15 (68.2)	0.972
Macula-off duration (days)	4.8 ± 2.3	4.6 ± 2.1	4.9 ± 2.4	0.721
Baseline BCVA (logMAR)	1.12 ± 0.34	1.08 ± 0.31	1.14 ± 0.36	0.567

### Morphological changes

### Central macular thickness

CMT differed significantly between groups throughout follow-up. At 12 months postoperatively, the silicone oil group showed persistently elevated CMT (287.4±42.3 μm) compared with both gas groups (SF6: 251.8±28.9 μm, p<0.01; C3F8: 248.2±31.7 μm, p<0.001). Normal age-matched CMT values range from 240-260 μm.

**Table 2:** Central macular thickness changes over time

Time Point	Silicone Oil (μm)	SF6 Gas (μm)	C3F8 Gas (μm)	p-value
1 month	324.6 ± 58.7	289.3 ± 45.2*	285.7 ± 41.8*	<0.001
3 months	308.2 ± 51.4	268.4 ± 38.6*	264.9 ± 36.2*	<0.001
6 months	297.8 ± 47.9	256.7 ± 32.1*	253.3 ± 33.7*	<0.001
12 months	287.4 ± 42.3	251.8 ± 28.9*	248.2 ± 31.7*	<0.001

*Significant difference compared to silicone oil group (p<0.01)

### Macular volume

Macular volume measurements paralleled the CMT findings. At 12 months, silicone oil eyes had significantly larger macular volumes (8.94 ± 1.23 mm³) than SF6 (8.21 ± 0.87 mm³, p<0.001).

### Structural complications

Epiretinal membrane (ERM) formation was more frequent in the silicone oil group (14/60, 23.3%) compared to gas-filled eyes (SF6: 3/38, 7.9%; C3F8: 2/22, 9.1%; p=0.042). Cystoid macular edema persisting beyond 6 months was observed in 13.3% of silicone oil cases versus 5.3% in SF6 and 4.5% in C3F8 groups (p=0.048).

### Perfusion changes

### Superficial capillary plexus

OCTA analysis revealed significant differences in superficial capillary plexus perfusion density between groups. At 12 months, silicone oil eyes showed reduced perfusion (42.8 ± 6.7%) compared with the SF6 (48.9 ± 5.2%, p=0.001) and C3F8 groups (49.3 ± 4.8%, p<0.001). Normal perfusion density ranges from 48-52% in age-matched controls.

**Table 3:** Superficial capillary plexus perfusion density

Time Point	Silicone Oil (%)	SF6 Gas (%)	C3F8 Gas (%)	p-value
1 month	38.2 ± 7.9	44.1 ± 6.3*	44.8 ± 5.9*	<0.001
3 months	40.6 ± 7.2	46.7 ± 5.8*	47.2 ± 5.4*	<0.001
6 months	41.9 ± 6.8	48.1 ± 5.5*	48.6 ± 5.1*	<0.001
12 months	42.8 ± 6.7	48.9 ± 5.2*	49.3 ± 4.8*	<0.001

*Significant difference compared to silicone oil group

### Deep capillary plexus

We observed similar patterns in deep capillary plexus perfusion. Silicone oil eyes showed persistently reduced perfusion density (39.4 ± 7.1%) at 12 months compared to gas-filled eyes (SF6: 45.2 ± 6.3%, p=0.002; C3F8: 46.1 ± 5.8%, p=0.001).

### Foveal avascular zone

The foveal avascular zone (FAZ) area was larger in silicone oil eyes (0.38 ± 0.09 mm²) than in gas tamponades (SF6: 0.31 ± 0.07 mm², p=0.003; C3F8: 0.30 ± 0.06 mm², p=0.001) at 12-month follow-up.

### Visual outcomes

Best-corrected visual acuity (BCVA) improved more in gas-filled eyes. At 12 months, mean logMAR BCVA was significantly better in the SF6 (0.34 ± 0.18) and C3F8 (0.31 ± 0.21) groups than in the silicone oil group (0.47 ± 0.25; p=0.008 and p=0.003, respectively).

**Table 4:** Visual acuity outcomes

Time Point	Silicone Oil (logMAR)	SF6 Gas (logMAR)	C3F8 Gas (logMAR)	p-value
1 month	0.78 ± 0.31	0.62 ± 0.27*	0.59 ± 0.29*	0.001
3 months	0.63 ± 0.28	0.49 ± 0.23*	0.46 ± 0.25*	0.002
6 months	0.54 ± 0.26	0.39 ± 0.20*	0.37 ± 0.23*	<0.001
12 months	0.47 ± 0.25	0.34 ± 0.18*	0.31 ± 0.21*	0.001

*Significant difference compared to silicone oil group

## Discussion

This study provides detailed quantitative analysis of macular morphological and perfusion changes following different endotamponade methods in retinal detachment repair. Our findings demonstrate significant differences between silicone oil and gas tamponades, with important implications for surgical decision-making and patient outcomes.

### Morphological considerations

The persistently increased central macular thickness observed in silicone oil eyes may reflect several factors. Silicone oil has been reported to induce inflammatory responses and interfere with normal retinal metabolism [5]. Direct contact between silicone oil and retinal tissue may disrupt the blood-retinal barrier, increasing vascular permeability and fluid accumulation. Silicone oil can also mechanically affect retinal architecture, potentially contributing to structural changes.

Gas tamponades demonstrated superior macular morphological recovery, with CMT values approaching normal ranges by 12 months. This finding aligns with previous studies suggesting that temporary tamponade agents allow for more natural healing processes without prolonged foreign body effects [6].

The significantly higher incidence of epiretinal membrane formation in silicone oil eyes (23.3% vs. 7-9% in gas groups) supports the hypothesis of increased inflammatory response associated with long-term silicone oil tamponade. This finding has important clinical implications, as ERM formation often necessitates additional surgical intervention and may compromise visual outcomes.

### Perfusion dynamics

OCTA analysis revealed profound differences in macular perfusion patterns between tamponade groups. The reduced perfusion density in both superficial and deep capillary plexuses in silicone oil eyes suggests impaired microvascular recovery [4,6]. Several mechanisms may explain this phenomenon:

1. **Direct toxic effects:** Silicone oil components may have direct cytotoxic effects on retinal capillary endothelium, leading to capillary dropout and reduced perfusion density.
2. **Mechanical compression:** The physical properties of silicone oil may create mechanical pressure on retinal vessels, compromising microcirculation.
3. **Inflammatory cascade:** Chronic inflammatory response to silicone oil may lead to capillary closure and reduced vascular density.
4. **Metabolic disruption:** Silicone oil may interfere with normal retinal metabolic processes, affecting vascular autoregulation mechanisms.

The enlarged foveal avascular zone in silicone oil eyes further supports compromised macular perfusion. FAZ enlargement has been associated with worse visual outcomes in various retinal conditions and can serve as a biomarker for macular ischemia [7].

### Clinical implications

These findings have several important clinical implications:

- **Surgical decision-making:** The superior morphological and perfusion outcomes with gas tamponades should be considered when selecting endotamponade agents, particularly in patients with good positioning compliance and uncomplicated retinal detachments. However, silicone oil remains the preferred option in cases of complex detachments with proliferative vitreoretinopathy, giant retinal tears, inferior breaks requiring prolonged tamponade, or in patients unable to maintain appropriate postoperative positioning.
- **Patient counseling:** Patients receiving silicone oil tamponade should be informed about the potential for prolonged macular changes and the possible need for closer monitoring and additional interventions [8,9]. Discussing the trade-offs between anatomical success rates and functional outcomes is essential.
- **Long-term Monitoring:** OCTA can serve as a valuable tool for monitoring macular perfusion recovery and identifying patients at risk for poor visual outcomes. Regular assessment of perfusion parameters may help guide the timing of silicone oil removal.
- **Treatment optimization:** The correlation between perfusion parameters and visual outcomes suggests that interventions aimed at improving macular perfusion may enhance functional recovery. Future studies should investigate adjunctive therapies to minimize perfusion deficits.

### Study limitations

We acknowledge several limitations. The retrospective design may introduce selection bias, although baseline characteristics were well-matched between groups. Surgeon preference, rather than randomization, guided the choice of tamponade agent, which may influence outcomes. The non-randomized design means silicone oil was more likely to be used in complex cases, potentially confounding the comparison [1,10]. Additionally, the study focused on morphological and perfusion parameters, and it did not include functional assessments such as microperimetry or multifocal electroretinography.

The relatively short mean follow-up of 14.2 months may not capture long-term changes, particularly in silicone oil eyes where the tamponade often remains in place for extended periods. Furthermore, we did not analyze the timing of silicone oil removal and its effect on macular recovery, which represents an important area for future investigation.

Future prospective randomized studies with larger sample sizes and longer follow-up periods would strengthen the evidence for optimal tamponade selection. Future research should investigate novel tamponade agents and adjunctive therapies to minimize macular changes.

## Conclusions

This study shows that, during retinal detachment surgery, the macula responds differently to endotamponade (gas (SF6 and C3F8) versus silicone oil), favoring gas.

- Normalization of central macular thickness is faster;
- Better preservation of macular perfusion density;
- Less frequent structural complications;
- Superior visual acuity recovery.

These findings support the use of gas tamponades, as appropriate, as well as the utility of multimodal imaging and quantitative macular assessment in the postoperative setting. The observed associations between perfusion metrics and visual outcomes may highlight OCTA’s potential utility as a prognostic tool in vitreoretinal surgery.

## Data Availability

The data that support the findings of this study are available from the corresponding author upon reasonable request.
